# 15-Year progression to liver cancer in the lack of treatment for lysosomal acid lipase deficiency: A case report

**DOI:** 10.1097/MD.0000000000030315

**Published:** 2022-09-02

**Authors:** Marlone Cunha-Silva, Eloy Vianey Carvalho de França, Clauber Teles Veiga, Raquel Dias Greca, Priscilla Brito Sena de Moraes, Daniel Ferraz de Campos Mazo, Elaine Cristina de Ataíde, Simone Reges Perales, Leonardo Trevizan Monici, Tiago Sevá-Pereira

**Affiliations:** a Division of Gastroenterology (Gastrocentro) – Department of Internal Medicine, University of Campinas (Unicamp), Campinas, Brazil; b Department of Surgery; University of Campinas (Unicamp), Campinas, Brazil.

**Keywords:** cholesteryl ester storage disease, hepatocellular carcinoma, liver cancer, lysosomal acid lipase deficiency, sebelipase alfa

## Abstract

Lysosomal acid lipase deficiency (LAL-D) is a poorly diagnosed genetic disorder characterized by the accumulation of cholesteryl esters and triglycerides in many tissues, leading to dyslipidemia and cardiovascular complications. In the liver, deposits are found within hepatocytes and Kupffer cells, generating microvesicular steatosis, progressive fibrosis, and cirrhosis. Sebelipase alfa is the target therapy which can improve laboratory changes and reduce the progression of liver damage, but this is not yet widely available.

**Patient Concerns:**

We are reporting a 15-year follow-up of a Brazilian man who was diagnosed with cirrhosis at age 43 and with LAL-D at age 53, but he has never been treated with sebelipase alfa for economic reasons. During the coronavirus disease 2019 (COVID-19) pandemic, he lost follow-up and missed three 6-month ultrasound exams for liver cancer screening.

**Diagnosis:**

At age 58, a remarkable deterioration in liver function was observed and he was diagnosed with hepatocellular carcinoma (HCC) outside the Milan Criteria (two nodules measuring 48mm and 25mm). Three other individuals with LAL-D and progression to liver cancer have been reported so far and none of them underwent enzyme replacement therapy: an 11-year-old girl with HCC, a 51-year-old male with cholangiocarcinoma, and a 21-year-old male with hepatocellular-cholangiocarcinoma. The latter had the same mutation in the gene LIPA as our patient, but a relationship between this variant and malignancies has not yet been established.

**Lessons:**

We emphasize how important is to treat LAL-D patients after diagnosis in order to avoid worsening liver function and progression to neoplasms. Untreated individuals should be considered at a higher risk but the most appropriate liver cancer screening program for this subgroup is still unknown.

## 1. Introduction

Lysosomal acid lipase deficiency (LAL-D) is a rare autosomal recessive genetic disorder associated with mutations in the gene LIPA, that encodes the enzyme lysosomal acid lipase (LAL).^[1]^ Its clinical spectrum ranges from an earlier-onset presentation (Wolman´s disease), with high mortality in the first 2 years of life, to a later-onset form, known as cholesteryl esters storage disease (CESD), which is characterized by a systemic accumulation of cholesteryl esters and triglycerides, including in hepatocytes and Kupffer cells. Patients with CESD may have serum lipid abnormalities, hepatosplenomegaly, elevated liver enzymes and progress to atherosclerosis, chronic liver disease and complications.^[1,2]^ Intestinal polyps leading to chronic diarrhea^[3]^ or bowel obstruction^[4]^ as initial manifestation have been reported. LAL-D is underrecognized and some patients present with cirrhosis and signs of portal hypertension at diagnosis.^[1,2]^

In affected adults, it is necessary to prevent and treat hepatic and cardiovascular complications, perform hepatocellular carcinoma (HCC) screening, and assess the need for liver transplantation. The specific approach consists of enzyme replacement (sebelipase alfa), which may improve the disease parameters such as transaminases, hepatomegaly, and dyslipidemia, reducing the progression of liver damage,^[5–7]^ but this therapy is not widely available.

There is a lack of data on the long-term follow-up of untreated LAL-D individuals, especially regarding liver cancer risk. We aim to report a 15-year progression to HCC in a patient who has never undergone enzyme replacement therapy. We obtained the subject’s consent to report the case. We will also highlight important similarities between this case and others previously published.

## 2. Case presentation

**Figure 1.**
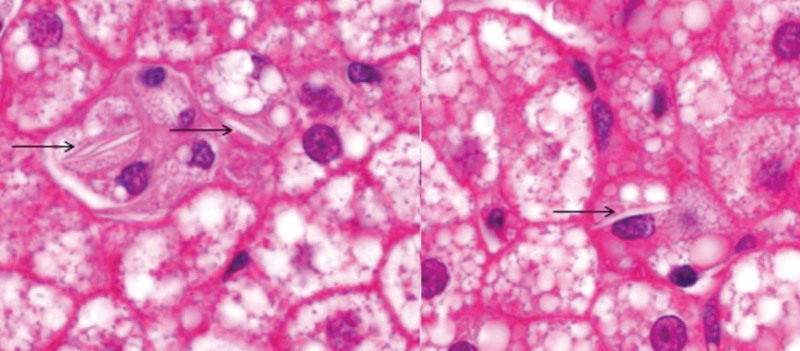
Histological findings when the diagnosis of lysosomal acid lipase deficiency was made (at age 53). Liver histology shows microvesicular steatosis and deposits of cholesteryl esters in Kupffer cells (arrows) (H&E 1,000x).

A 43-year-old Brazilian man was referred to our tertiary center with asymptomatic splenomegaly and thrombocytopenia detected on routine exams. He was diagnosed with cirrhosis Child-Pugh A but the etiology of liver disease was elucidated at age 53, when hepatic histology showed microvesicular steatosis and cholesteryl esters deposits in hepatocytes and Kupffer cells (Fig. 1). Details of the case and all steps for diagnosing LAL-D have already been published.^[1]^

His LAL activity was undetectable using the blood spot test (at Seattle Children´s Hospital) and a variant mutation in the gene LIPA was observed *(allele c.386A > G homozygous p.H129R*).^[1]^ He has been on atorvastatin, vitamin D3, hydrochlorothiazide, and carvedilol and has not started the enzyme replacement for economic reasons, as this therapy is not available in the Brazilian public health system.

**Figure 2.**
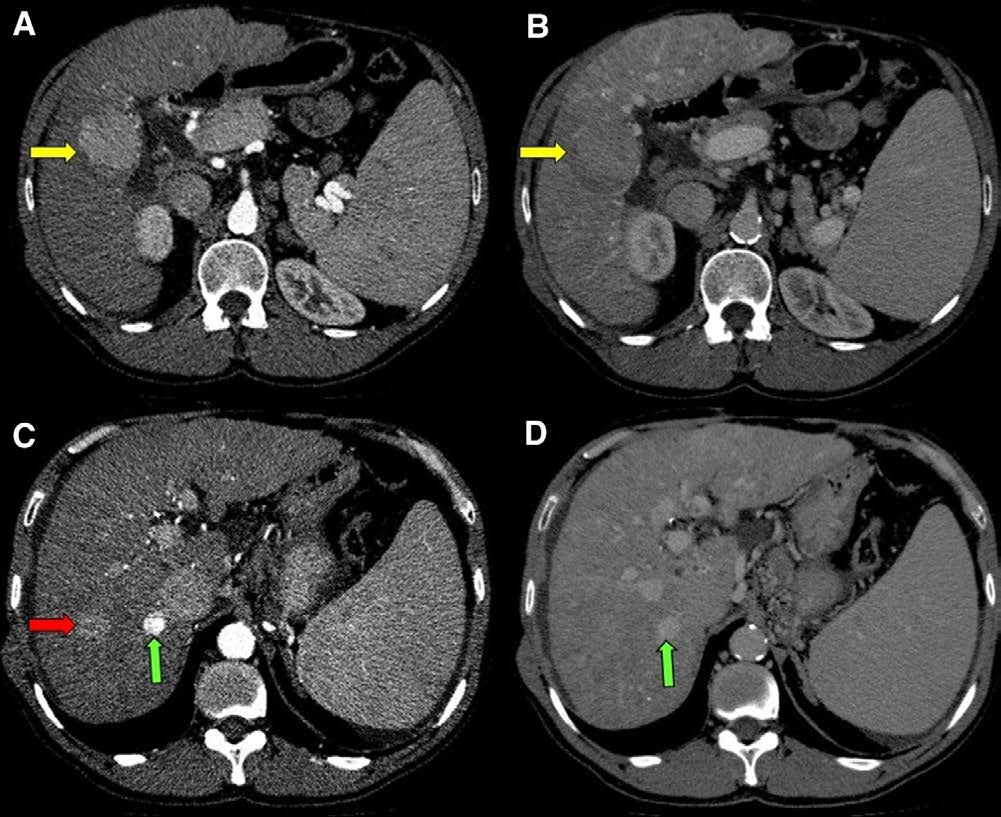
Cross-sectional computed tomography of the abdomen. In segment V of the liver, there was a contrast-enhanced nodule in the arterial phase (A, yellow arrow) with washout in the portal phase (B, yellow arrow), measuring 48mm in largest diameter (LI-RADS 5). In segment VII, another contrast-enhanced nodule in the arterial phase was evidenced (C, red arrow) with no typical washout in the portal phase (D), measuring 25mm in largest diameter (LI-RADS 4). There was also a nodule measuring 14mm in segment VII with arterial enhancement (C, green arrow) and high density in the portal phase (D, green arrow), which was previously characterized as hepatic hemangioma (LI-RADS 1).

During the coronavirus disease 2019 (COVID-19) pandemic, he lost follow-up and missed three 6-month ultrasound exams for HCC surveillance. The medical staff contacted him to return to outpatient follow-up. At age 58, a remarkable worsening in laboratory tests and liver function scores was observed, even in the absence of symptoms. On abdominal ultrasound, hepatic nodules were evidenced, and further contrast-enhanced computed tomography showed radiological features of HCC: one lesion measuring 48mm in segment V with arterial enhancement followed by portal-venous washout (Liver Imaging Reporting and Data System (LI-RADS) 5) and another tumor measuring 25mm in segment VII with arterial enhancement but lack of typical washout (LI-RADS 4) (Fig. 2).

Chest tomography and bone scintigraphy did now show tumor spread. Serum alpha-fetoprotein was 42.3 ng/mL (normal value < 7 ng/mL). Table 1 shows his main laboratory tests and liver function scores at the diagnosis of cirrhosis (43y) and HCC (58y). The Barcelona Clinic Liver Cancer (BCLC) staging was B, but outside the Milan Criteria,^[8]^ so the medical team decided to perform transcatheter arterial chemoembolization and subsequent inclusion on the liver transplant waitlist by downstaging.

**Table 1:** Characteristics of the reported case: at the diagnosis of cirrhosis and HCC (hepatocellular carcinoma).

Exams at the diagnosis of:	cirrhosis (43y)	HCC (58y)	Normal range
Hemoglobin (g/dL)	16	13	14–18
Leucocytes (x10^3^/mm^3^)	4.57	3.01	4–10
Platelets (x10^3^/mm^3^)	103	34	150–400
ALT (IU/L)	76	49	<50
AST (IU/L)	63	81	<50
ALP (IU/L)	131	215	<129
GGT (IU/L)	81	83	<60
Total bilirubin (mg/dL)	1.26	3.34	0.3–1.2
INR	1.10	1.96	<1.25
Albumin (g/dL)	4.8	3.3	3.5–5.2
Alpha-fetoprotein (ng/mL)	5	42.3	<7
Child-Pugh score	A 5	B 9	–
MELD score	8	19	–
MELD-Na score	10	20	–

AST = aspartate aminotransferase, ALT = alanine aminotransferase, ALP = alkaline phosphatase, GGT = gamma-glutamyl transferase, INR = international normalized ratio, MELD = model for end-stage liver disease, Na = sodium.

## 3. Discussion

Clinical trials with sebelipase alfa in adult patients have shown encouraging outcomes, both in terms of safety and improvement of laboratory tests.^[7,9,10]^ Despite being approved for prescription since 2017 in Brazil,^[11]^ this is not yet available in the Brazilian public health system, so our patient could not start it even after a judicial request.

Our patient was followed up for 10 years as having cryptogenic cirrhosis and for 5 years with the diagnosis of LAL-D. In the lack of a specific therapy for the etiology of cirrhosis, worsening liver function and laboratory changes were expected, as shown in Table 1. It is known that treating the etiology of liver disease is essential to delay or prevent the progression of hepatic fibrosis. This reduces the risk of hepatic decompensations and malignancies, which decreases the need for liver transplantation and mortality rates.^[12]^

There is scarce data on liver cancer in LAL-D patients. Although the cholesteryl esters and triglycerides are deposited in hepatocytes and Kupffer cells, distinct progression to malignancies has been reported: a girl with HCC (11y),^[13]^ a man with cholangiocarcinoma (51y),^[14,15]^ and a man with hepatocellular-cholangiocarcinoma (21y).^[16]^ The latter had the same mutation in the gene LIPA as our patient (allele c.386A > G homozygous p.H129R). We do not know whether this is a coincidence or this mutation is associated with a higher risk of liver cancer. An experimental model suggested that endothelial cells in LAL-D mice facilitated in vivo tumor angiogenesis, growth, and metastasis, largely by stimulating tumor cell proliferation, adhesion, and transendothelial migration via increased expression of interleukin-6 and monocyte chemoattractant protein 1.^[17]^ The role of PPAR-alpha and mTOR pathways for tumor stimulation in LAL-D hepatocytes has also been studied, but no specific mutation in the gene LIPA has so far been strongly associated with malignancies.^[18]^

The main features of reported LAL-D patients with cancer are shown in Table 2. It is essential to highlight that there are no reports of liver neoplasms in LAL-D patients on long-term sebelipase alfa.^[19]^ In contrast, none of those subjects who progressed to liver cancer underwent the enzyme replacement, which suggests that the use of sebelipase alfa could reduce the incidence of liver malignancies in this subgroup. Beyond the lack of specific therapy for LAL-D, our patient missed the liver cancer screening, which certainly contributed to the diagnosis of HCC outside the Milan Criteria.^[20,21]^

**Table 2:** Characteristics of reported patients with lysosomal acid lipase deficiency and progression to liver cancer.

Age	Gender	Characteristics	Ref.
11y	F	HCC, *unreported mutation*	^[13]^
51y	M	Cholangiocarcinoma,*heterozygote for G934A and DeltaC(673-5*)	^[14,15]^
21y	M	Hepatocellular-cholangiocarcinoma, *c.386A > G homozigous p.H129R*	^[16]^
58y	M	Radiological features of HCC, *c.386A > G homozigous p.H129R*	[*]

The patient with hepatocellular-cholangiocarcinoma had the same mutation as ours. The last one is our case [

* ].

HCC = hepatocellular carcinoma, F = female, M = male. Ref. = reference.

An effective approach to screen HCC in patients at a higher risk is 6-month abdominal ultrasound accompanied or not by alpha-fetoprotein dosage.^[20,21]^ Surveillance for cholangiocarcinoma remains a challenge as there is no consensus among experts. The combination of cancer antigen 19.9 (CA19.9) dosage and magnetic resonance cholangiography every 1 or 2 years seems to be the most common strategy, however, this is based on studies involving mainly patients with primary sclerosing cholangitis.^[22]^

In conclusion, liver malignancies in LAL-D patients are rare and may range from cholangiocarcinoma to HCC. It is possible that the use of sebelipase alfa can reduce the incidence of neoplasms in affected patients, therefore untreated individuals should be considered at a higher risk, but the most appropriate strategy for cancer surveillance is not yet established.
